# Medical Opinion and Movement

**Published:** 1907-02-23

**Authors:** 


### Medical Opinion and Movement.

Dr. Chalmers Watson, to whose work on the effects of a meat diet on rats we have already made reference in a previous issue, has evolved a theory, largely based upon this work and the changes he has observed in the thyroids of rats fed on meat, by which he seeks to establish an antagonism between the "gouty" diathesis and the tuberculous diathesis. He suggests that the so-called gouty diathesis is dependent upon an excessive activity of the thyroid gland, and requires a limited supply of meat in the diet. On the other hand, subjects predisposed to tuberculosis are, he supposes, deficient in thyroid activity, and require a liberal diet with a full proportion of proteid. He cites many interesting clinical observations in support of this theory, but the facts hardly warrant any such general conclusions. The work on which Dr. Chalmers Watson is engaged is full of interest, and any glimmer of light which he may succeed in throwing upon the much-vexed questions of diet will be welcome, but too hasty assumptions will not tend to advance any real knowledge on the subject.

Unwritten law is always more liable to transgression than a code in black and white, and may often be broken from ignorance rather than wilfulness. It is well, therefore, that the established customs of the profession in regard to consultations should be definitely and clearly set forth by some responsible authority of the profession. The central ethical committee of the British Medical Association has undertaken this duty, and has drawn up a series of propositions in regard to the ethics of medical consultations, which, if adopted by the Association, should prove a useful code of rules for the guidance of the profession. In these recommendations the various questions, which naturally arise in connection with consultations, are considered in detail: The duty of consultation, choice of consultant, conditions justifying refusal to meet a practitioner in consultation, method of procedure in consultation, and so forth. The committee further enumerates those cases in which it is specially the duty of the practitioner in attendance to endeavour, if possible, to obtain a second opinion. The term "consultant" is defined as the designation of any practitioner who is called in to advise on any particular case, but is not in continuous attendance. The question of a formal recognition of "consultants" as a class of medical practitioners is considered, and advantages resulting therefrom are pointed out, but no definite recommendations are made on the matter.

A serious difficulty is arising in regard to the ever-increasing number of synthetic drugs which are continually appearing in the market, a certain proportion of which at any rate make good their claim to therapeutic value, and form a necessary part of the physician's armamentarium. These drugs are often highly complex organic compounds, and the systematic names by which they are known to chemists are of such a length as to preclude their use for prescribing purposes. It is necessary, therefore, to give them some synonym for practical use, and the manufacturer has the right under the Trade Marks Act to register the name he chooses to apply to the drug. This registration precludes any other maker from supplying the drug under the same name. Such a drug, therefore, usually becomes known by the name registered by the maker first in the field, or by the firm who is most successful in pushing it upon the notice of the profession and the public. Other makers, however, soon follow, but are compelled to adopt another name for the same drug. In consequence there is inevitable confusion from a multiplicity of synonyms. To take an example: urotropine is synonymous with aminoform, cystamin, cystogen, formin, metramine, urisol, uritone, naphthamine, and vesalvine. In the "Chemists' Annual" for 1906 a glossary of these synonyms is given, and an attempt is made to introduce some uniformity in the nomenclature of these drugs. It is suggested, for instance, that all local anæsthetics shall end in-caine. It is highly necessary that some authority should take upon itself the duty of giving an unregistered synonym to any of these drugs, which become of general utility, so that they may be ordered and prescribed under that name to the exclusion of all other proprietary terms.

The treatment of rodent ulcer with *x*-rays has been attended with such favourable success, that it will not readily be abandoned in favour of any other new treatment. Dr. H. Lewis Jones claims to have successfully treated several cases by the application of zinc ions, by means of electrolysis of zinc sulphate solution with the constant current. This treatment has the advantage over *x*-rays or radium that it is much simpler and requires a less complicated and less expensive apparatus and less technical skill on the part of the operator. He has treated nineteen cases, and, with the exception of five, the results have been successful. Of these five, in one the treatment has been abandoned by the patient, the remaining four are still under treatment, and in only one of these does Dr. Jones appear to have any apprehensions as to the result. The method of procedure was initiated by Dr. S. Leduc of Nantes. An ordinary medical continuous current battery with galvanometer is used. The negative pole is connected with a pad electrode and applied to some convenient part of the patient. The positive pole is connected with a zinc rod covered with a small pad of lint soaked in a 2 per cent. solution of zinc sulphate. This is applied to the ulcer and the current slowly turned on to 5 or 10 milliampères, according to the size of the electrode. In this way zinc ions are carried by the current into the tissues of the ulcer. One application of ten or twelve minutes is in many cases sufficient to effect a cure. In certain cases with induration and outlying nodules Dr. Jones resorts to needling with a zinc needle, and these require more prolonged treatment. The author suggests that there may be a wider field for this form of "ionic" treatment. He has not yet succeeded in finding a suitable medicament for the electrolysis of lupus.

